# A Process Evaluation of a Mobile App for Medical Students Aimed at Increasing Resilience and Decreasing Stigma in Mental Health

**DOI:** 10.7759/cureus.63054

**Published:** 2024-06-24

**Authors:** Natalie M Fadel, Alexis Stoner, Krisdaniel Berreta, Angela Wilson, Lindsey M Ridgeway, Duke Biber, Harold R Garner

**Affiliations:** 1 Psychiatry and Neuro-Behavioral Sciences, Edward Via College of Osteopathic Medicine-Carolinas Campus, Spartanburg, USA; 2 Epidemiology and Public Health, Edward Via College of Osteopathic Medicine-Carolinas Campus, Spartanburg, USA; 3 Psychiatry, AtlantiCare Regional Medical Center, Atlantic City, USA; 4 Data Quality Analysis, Mayo Clinic, Rochester, USA; 5 Student Affairs, Edward Via College of Osteopathic Medicine-Carolinas Campus, Spartanburg, USA; 6 Health Sciences, James Madison University, Harrisonburg, USA; 7 Bioinformatics, Proposed Illinois College of Osteopathic Medicine (IlliniosCOM) at The Chicago School, Chicago, USA

**Keywords:** psychiatry and mental health, behavioral health, suicide prevention, mental health apps, mobile health, mental health, medical students

## Abstract

Objective: The purpose of this pilot study was to conduct a process evaluation of a mental health and wellness mobile health (mHealth) application for medical students designed to increase resilience and decrease mental health stigma.

Methods: The customized application, MindfulMEDS, was developed with peer-focused interactive modules specific to medical students within an existing system called Sharpen^®^. The Sharpen^®^ system contains an extensive library of didactic and experiential mental health and wellness content built specifically to promote evidence-based protective factors for resilience. A mixed-methods approach including surveys and focus groups assessed participant resiliency, perception of mental health within the context of medical school, and evaluation of the app. Assessments were conducted at baseline (n = 66), six months (n = 30), and one year (n = 24). Demographic information was collected once at baseline as a part of the initial survey.

Results: A total of 215 users were registered in MindfulMEDS, consumed 83 courses, and engaged in 1,428 “connect clicks” to community resources and crisis-response supports. Resilience levels did not change significantly between surveys; however, a significant decrease in the perception of mental health stigma associated with utilizing mental health resources was observed. Focus group participants (n = 11) reported the screening tools to be useful, encouraged expansion, and suggested additional reminders to access the app to increase engagement.

Conclusion: Findings of this pilot study demonstrate the feasibility of implementing MindfulMEDS (an mHealth app focused on mental health and wellness) among medical students. Students found the app experience valuable, accessed mental health screeners embedded within the app, utilized the app to seek help, and engaged with the app to learn more about mental health. There was also a decrease in mental health stigma observed during the course of the study. Based on these results, we propose that medical schools incorporate mobile-based technology into their mental health support programs.

## Introduction

The environment in which medical students train can be characterized by the presence of multiple stress factors, such as academic workload, witnessing of patient suffering, and sleep deprivation. Historical literature indicates the medical school setting can predispose medical students to increased psychological distress, thus creating a pathway from experiencing burnout to lower levels of empathy and mental impairment as they progress through the curriculum [1,2].

The mental health of medical students has become a topic of interest in recent years and remains in the spotlight [3-5]. Medical students experience higher rates of depression, anxiety, and suicidal ideation, compared to the average population [6-8]. This epidemic of mental illness among medical students is present in schools across the United States and extends internationally [9,10]. Despite the known heightened risk for mental disorders, medical students, residents, and attending physicians are still reluctant to reach out for help or treatment. In an anonymous survey of 1,048 academic physicians, 12% reported moderate to severe depressive symptoms in the last two weeks, and fewer than half reported that they were likely to seek treatment for a mental health concern. Stigma and access to treatment, specifically difficulty with scheduling, were major barriers to seeking treatment [11]. Another study found that personal stigma, having one's own stigmatizing beliefs, was an explicit barrier to the use of mental health services by 30% of first- and second-year medical students experiencing depression. In addition to stigma, other barriers to treatment included lack of confidentiality and fear of documentation in their academic record [12].

The relationship between stigma and suicide is complex, as it can be bidirectional, direct, and indirect. For example, a person suffering from depression who fears consequences due to stigma and does not seek treatment has an increased risk of suicide, and someone who has a history of suicidality may be stigmatized and therefore also at higher risk [13]. With rates of suicidal ideation being significantly higher in this population, there is a critical need for further research to prevent these mental disorders and treat them [14].

With the ever-increasing usage of mobile-based technology, such as smartphones and tablets, young adults have a higher likelihood of becoming more proactive and informed of choices for their mental health care, especially when prompted and if information is made easily accessible [15]. Data show that when provided a stress-reducing product via a mobile application, students experienced a significant reduction in stress, while also improving mindfulness in their daily behaviors [16]. Research also shows potential for implementing mental health and resiliency training for those suffering from illnesses such as schizophrenia and depression [17,18]. As a part of the ongoing effort to cultivate wellness in medical students, an evidence-based mobile application, MindfulMEDS, was created to support stress management, healthy relationships, and improving coping skills. This app offers students a confidential and private mechanism for access to information regarding mental health and wellness resources, accessible behavioral health treatment options, and a mechanism to connect to support on an instantaneous basis. Furthermore, the peer-to-peer content embedded within the app aims to decrease stigma and normalize discussions around mental health in medical school. 

The current pilot study sought to conduct a process evaluation of the MindfulMEDS mHealth application for medical students through a mixed-methods approach. The MindfulMEDS app was designed using original content as a contemporary approach to support mental health issues for medical students. This process evaluation will focus on the utility, acceptance, and value of the application by medical students. It will also inform future implementation and evaluation of the MindfulMEDS app on medical students’ resilience, access to mental health resources through engagement with the app, and perception and understanding of mental health.

## Materials and methods

MindfulMEDS application development

MindfulMEDS, a mental health and wellness app for medical students, was developed in partnership with Resiliency Technologies (RT), a leader in the mental health technology field. RT has developed the Sharpen® system to improve the shared protective factors for suicide, trauma, and mental disorders, including mental health literacy (MHL), social-emotional learning (SEL), and mindfulness-based stress reduction (MBSR) for a variety of populations. Sharpen® employs a HIPAA (Health Insurance Portability and Accountability Act)- and FERPA (Family Educational Rights and Privacy Act)-compliant communication network, which assists providers and mission-driven organizations to increase outcomes in engagement and compliance following best practices with mental health screening and education with their constituents. The Sharpen® system was created over the course of 15 years through interdisciplinary research collaborations and community-based documentary film projects highlighting the power of peer stories of strength and resilience [19]. The videos contained within Sharpen® modules were developed using the same formula that Hussa and Farrell designed for Bloomberg Philanthropies-funded public art project called “Video Village” - one of eight installations included in “Seeing Spartanburg In a New Light” where over 100 residents from public housing developments engaged in sharing video testimony to increase community resilience [20].

MindfulMEDS is a customized application of the Sharpen® system, developed to help connect students to mental health literacy trainings, community resources, crisis response support, emotional wellness, and coping skills-builders assisting in managing the stress of medical school. The system aims to increase resiliency and overall wellness, which can help combat increased rates of depression, anxiety, and suicidal ideation [21-24]. Medical students and physicians experience these conditions at greater levels as compared with the general population [6-8,25-27]. The development of the MindfulMEDS application occurred over a one-year period in which RT founders collaborated closely with team members at the Edward Via College of Osteopathic Medicine - Carolinas (VCOM) to build and customize a version of the Sharpen® service specific to the interests, needs, and unique engagement variables associated with medical students. Several cycles of development and formative evaluation were conducted and included product testing with medical students to ensure reliable and accurate feedback. This process was essential to ensure the success of MindfulMEDS for the medical student population.

The Sharpen® system includes a core library of vetted psychoeducational content, to which 10 peer-focused interactive modules were added specifically to the health and well-being of medical students. Sharpen®’s peer-to-peer, documentary film content library was built using eight evidence-based protective factors shown through research to improve resiliency [28], while offering providers and patients a toolkit to assist in recovery from an array of mental disorders. Example topics comprising the 550 core Sharpen® MHL, SEL, and MBSR modules include childhood trauma, ACEs (adverse childhood experiences), toxic stress, mindfulness interventions, test anxiety, improving coping skills, healthy relationships, improving communication skills, suicide prevention, substance use prevention, disordered eating prevention, professionalism, and mental health literacy. These core modules are housed under four primary protective factor categories, titled “Cope, Thrive, Nourish and Heal” within Sharpen®’s Discovery library (Table 1). In addition, the app provides the unique service of connecting students to resources whether they are at our main campus or at clinical site locations.

**Table 1:** Sharpen® system design.

Title	Primary protective factor	Secondary protective factor	Tertiary protective factor
Cope	Social-emotional learning (SEL)	Healthy coping skills	Improving community connectedness
Thrive	Mindfulness-based stress reduction (MBSR)	Stress management	Financial health and literacy
Nourish	Evidence-based dietetics models for disordered eating prevention	Body esteem and body satisfaction	Improving healthy relationships
Heal	Mental health literacy (MHL)	Psychoeducation on primary mental disorders	Suicide prevention

**Figure 1:**
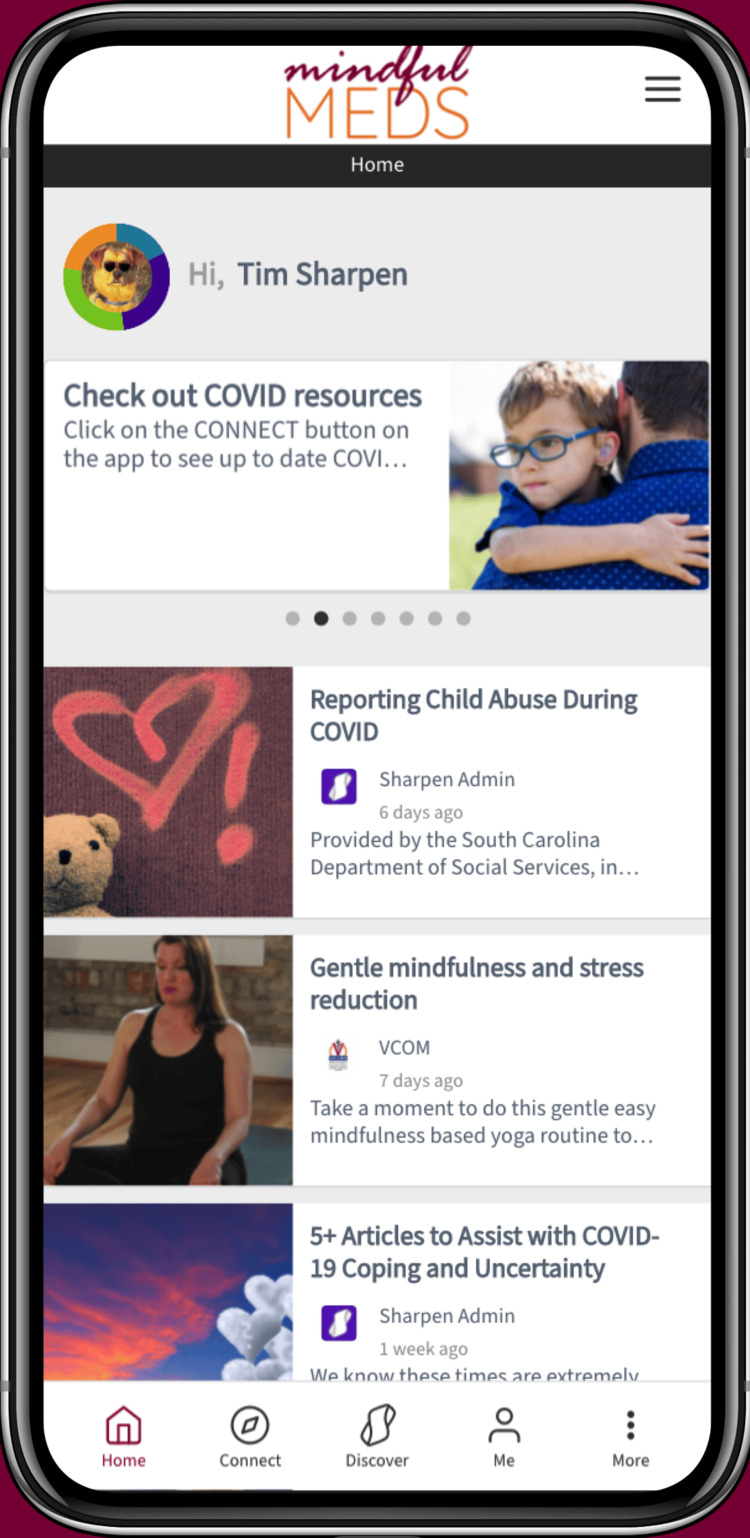
MindfulMEDS app feed example.

**Figure 2:**
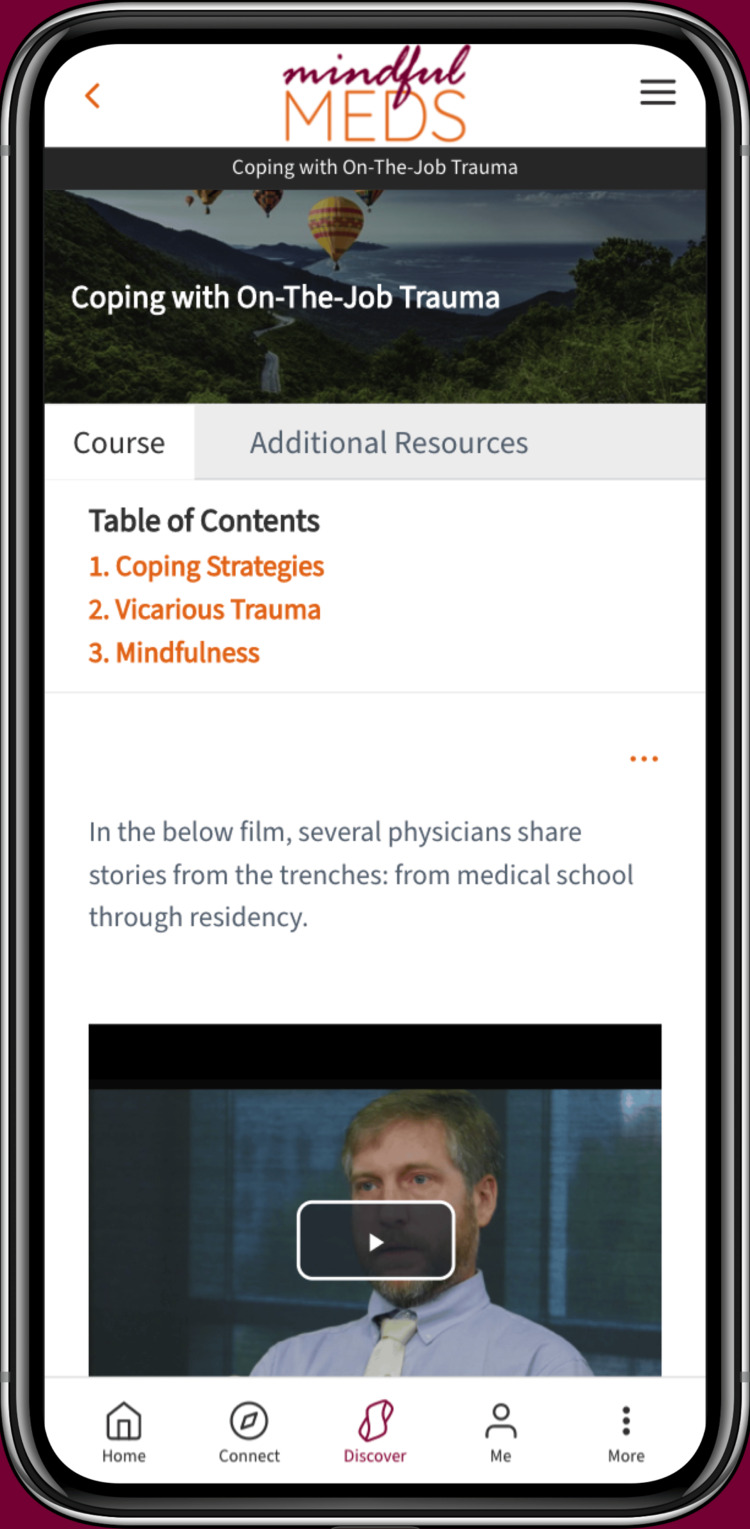
MindfulMEDS app module example under the "Discover" button.

MindfulMEDS users can explore protective content in varying areas of the app including the feed, where shorter, customized posts by the app managers can be deployed (Figure 1): the connect button, where students access community-based resources, treatment resources, and “Get Help Now” information, Mindsharpeners (quick mindfulness activity cards), mental health screenings and crisis response support and the Discover button where students access a library of modular content (Figure 2).

Participant recruitment

A convenience sample of students enrolled at VCOM Carolinas campus was taken during the 2019-2020 academic year. VCOM enrolls approximately 170 students on each of four campuses each year. First- and second-year VCOM students were given a brief in-person presentation on campus, and third- and fourth-year students were given the same information via email to introduce the app and invite them to participate in the study. Students from all four classes were eligible to participate. Students were offered entrance into a lottery for an Amazon gift card for their participation. The research team emphasized that all students would be given access to the app regardless of participation in the study. Follow-up emails were sent to remind students of the technical support available as they began to navigate the app. VCOM, on average, matriculates approximately 57% female students to 43% male students with some variability between academic years. Students have a mean age of 24 years at the time of matriculation, and most are from the Appalachian region of the United States with a majority from North Carolina and South Carolina.

Study design 

Students who chose to participate in the study completed a Qualtrics, Provo, UT pre-survey delivered via the MindfulMEDS application. The survey included questions regarding demographics (e.g., age, medical school year, and gender identity), resiliency, connectedness, and perception of mental health. Space was provided for open-ended feedback on the MindfulMEDS app in the post-surveys. Resilience was assessed using the Brief Resilience Scale, a validated measure focused on an individual’s ability to bounce back from stress and adversity [29]. The BRS is a brief, single-factor instrument with three positively worded items and three negatively worded items to minimize response bias. The BRS is scored by reverse coding items 2, 4, and 6 and finding the mean of the six items. Each item is assessed on a Likert scale (i.e., 1 = strongly disagree, 2 = disagree, 3 = neutral, 4 = agree, 5 = strongly agree). In a recent review of resilience scales, the BRS was noted to have the most satisfactory psychometric properties (good Cronbach's alpha of 0.80) and was said to be one of the most frequently used resilience scales in a total of 25 scales [30]. Questions regarding connectedness and perception of mental health were also assessed on a five-point Likert scale. The following are example questions: “There is at least one faculty or staff member at VCOM whom I feel comfortable talking to about things that are bothering me,” “I know how to access mental health resources available to me,” and "There is a stigma associated with utilizing mental health resources." The survey was administered at baseline, at six months follow-up, and one-year follow-up.

In addition, usability metrics were collected throughout the one-year study and provided by Sharpen®, which included the number and category of module consumption and number of engaged users (individuals who registered and accessed the app). The Sharpen® system allows for de-identified data gathering showing the aggregate activity utilized and the content and protective factors explored. Utilizing analytics to evaluate digital mental health interventions has become a growing trend [31]. Using Elastic’s Kibana (Elastic, Mountain View, CA) dashboards linked to the Sharpen® database, Google Analytics, and the Sharpen® CMS, the findings from MindfulMEDS include aggregated, de-identified data collected from registered users.

Two different focus groups were conducted (the first at six months and the second at one year) to assess the usability and student perceptions of the MindfulMEDS app to inform future versions and implementation. A structured interview was conducted that included questions regarding general feedback, level of satisfaction, and suggestions for the development and improvement of MindfulMEDS. Students from all four VCOM classes were recruited to participate in these focus groups and informed consent was obtained.

Data analysis

Descriptive analyses were performed on student demographics. Differences between survey responses were compared using a chi-square test, and Fisher’s exact test was used when sample sizes were small, or the data were unequally distributed. Due to attrition rates, there were not enough individual pre/post to pair for analysis. We instead compared groups and the five-point Likert scores were analyzed as categorical data. A p-value less than 0.05 was considered statistically significant. Focus groups were voice-recorded and transcribed. Participants were assigned a numerical code to ensure anonymity. The transcriptions were coded and thematically grouped using qualitative analysis methodology.

## Results

A total of 66 students consented to participate in surveys and provided demographic information at baseline. Of the participants, 63.6% identified as female (n = 42), 33.3% as male (n = 22), 1.5% as transgender female (n = 1), and 1.5% as transgender male (n = 1). In terms of race, 77.3% identified as White (n = 51), 10.6% as Asian (n = 7), 6.1% as other (n = 4), 3% preferred not to answer (n = 2), and 1.5% identified as African American (n = 1) and native Hawaiian/Pacific Islander (n = 1). At six months, 30 students filled out a post-survey, and at one year, 24 students completed the post-survey. Thirteen students completed all three surveys. Of those who completed all three surveys, most (84.6%) were White, had a median age of 23, and were divided equally between males (53.9%) and females (46.2%). In terms of school classification, 87.9% were osteopathic medical student (OMS) year 1 (n = 58), 7.6% were OMS year 2 (n = 5), 1.5% were OMS year 4 (n = 1), and 3% preferred not to answer (n = 2).

MindfulMEDS user activity

**Figure 3:**
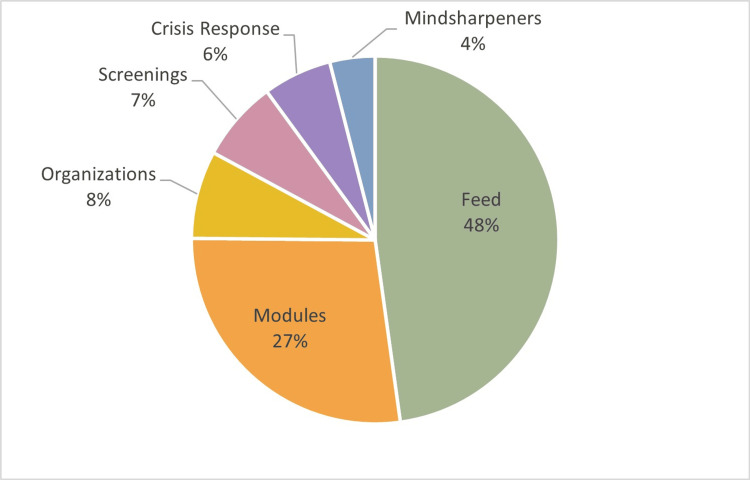
Overall page views by category.

While only 66 students initially consented to participate in the surveys, a total of 215 users were registered in MindfulMEDS during the study period. Over the course of the one-year study period, users consumed 83 courses, and 173 mini-courses were deployed in the Sharpen® feed and engaged in 1,428 “connect” clicks to community resources and crisis-response supports. We did not track individual usage, so there is likely great variability in how much content each registered user consumed. The highest pageview counts on MindfulMEDS were accessed in the feed, while study tips, mindfulness-based stress reduction, and time management strategies held MindfulMEDS users’ attention for the longest average length of time within the various sections of the app. Figure 3 provides details on what content received the most pageviews by percent.

The feed contained over 173 mini-courses and posts that were deployed throughout the course of the study. The post content was variable and included topics such as crisis response information, general information about the app, and helpful tips from fellow medical students. Measured in pageview clicks, the “Get Help Now” button and “About this App” received the majority of the pageviews, with medical student and community athletic-focused feed items being the most utilized. Within the app, the “Get Help Now” button was clicked 62 times, and the “Crisis Line” button was clicked 14 times. “Get Help Now” connects students to localized, sliding-scale resources for mental health treatment and, as the name implies, includes immediate connection to licensed psychotherapists both within the medical school and outside (such as local substance use and mental health treatment providers). Organizations include local resources for extracurricular interests or needs such as community outreach, outdoor recreation, art, music, and sports.

Modular content accessed through the Discover button in the app was divided into the primary protective factor categories outlined above. According to MindfulMEDS user pageview counts, the most consumed modular content was in mindfulness-based stress reduction (124 page views), followed by mental health literacy (69 page views), and eating disorder/prevention (54 page views). The Crisis Response items accessed on the app included the “Get Help Now” resources and the National Suicide Prevention Lifeline. The top mental health screenings accessed on the app included ADHD self-report assessment and depression test. Users on MindfulMEDS were connected to 12 community-based organizations through the community connections (CONNECT) button. Each organization has a descriptive page with a connection to services and additional resources. The organizations included in the app included outdoor activities/sports, mental health organizations, and community-based organizations with the most accessed being community and outdoor resources to support medical student community engagement. Finally, MindfulMEDS users accessed a variety of Mindsharpeners (quick mindfulness activities that include journal writing techniques and positive affirmations), most frequently empowering quotes, such as “Start where you are. Use what you have. Do what you can" (by Arthur Ashe, tennis star.

Focus group feedback

A total of five students participated in the first focus group at six months, and six students participated in the second focus group at one year. Overall, students reported that they “like the app.” Students found the screening tools helpful and suggested this area be expanded further to include more options. For example, one student mentioned: *I remember a few years ago, I was going through a really rough time, and I wasn’t sure if I was depressed or not. And then so I was like, should I go see someone to talk about it or not? And so, I was working at a clinic at that time, and I knew we had the PHQ *(referring to Patient Health Questionnaire depression screening) *forms or questionnaire or something like that. So, I was wondering would it be a good idea to have, some type of form like that available…like hey, if you’re feeling this way and you’re unsure, do you want to do this survey to see if you’re depressed?*

Although important and desired, some students expressed difficulty in using the screeners due to a lack of functioning: *It just doesn’t pull up, like I get to where it says anxiety screener on the bar at the top. But no window ever opens down on the main page. I think it was working for all the others. But it’s just the anxiety one will not pull up for me. *Participants from both focus groups reported some difficulty with navigation and suggested making navigation clearer and more user-friendly. These difficulties were due to internal technical errors, which were fixed successfully subsequent to the focus groups.

Students were interested in receiving additional notifications and believed regular notifications regarding added content and/or reminders to access the app would yield more engagement. For example, weekly emails regarding app content and push notifications via text were unanimously supported by focus group members. One student exhibited this: *But I agree, I think the notifications would be nice. Just as a reminder that, you know, it’s here. And there’s a new tip for you or something.*

Participants were interested in topics such as stress management, nutrition, exercise, and location-specific information related to recreational activities, fitness, events, and resources. For example, one student stated,* I’m a first year and I had access to it for probably like, six months or so. Over the last six months, I probably used it for a couple of hours. I’ve just used the resources to see stress management and ideas to deal with stress.*

One student reported using the app to share content and resources with patients in a clinical setting, and other students indicated they would be interested in using the app for this purpose as well: *I do actually think it’s really helpful to go through the stuff (MindfulMEDS) myself too and then be able to apply it to patients. I think even once some of you guys get out into the third year and into the clinic, you’ll see this is the kind of stuff that your patients like. First of all, they’re anxious to see you, but also like they’re just anxious about what’s going on with themselves. So obviously, the better that you feel right, the more help you can give other people. So, I think it’s nice for me to go through this to kind of make sure that I’m checking in with myself and doing okay. It does help me be better for my patients too. So, I enjoy using the app and kind of seeing what I can find on there.*

Outcome variables Brief Resilience Scale (BRS)

From baseline to six-month follow-up, there was a 55% attrition rate, meaning only 30 participants completed the follow-up compared to 66 participants at baseline. Furthermore, only 24 participants completed the surveys at one-year follow-up. That said, there were no significant differences found in resilience levels as reported in the BRS between the baseline and six-month survey (p = 0.42), one-year follow-up surveys 2 to 3 (p = 0.87), and one-year follow-up comparing surveys 1 and 3 (p = 0.23). We did, however, see a consistent pattern in levels of resilience at baseline, six months, and one year. Results show that the majority of the students who completed the BRS scored in the “Normal resilience” range at each stage, with the next highest percentage of students scoring in the “Low resilience.” Of those who completed the survey, the lowest number of students scored in the “High resilience” range.

Mental health stigma and connectedness to the school community

There were significant differences found overall (p = 0.011) and between the pre-survey and six-month follow-up (p = 0.020) in students’ response to feeling students at VCOM help each other even if they are not friends. Similarly, significant differences were found between surveys in students reporting they feel there is a faculty member they are comfortable talking to about things that are bothering them (p = 0.003), they spend time with at least one peer outside of class time (p=0.009), and they know how to access mental health resources available to them (p = 0.005). It is notable that these differences are between groups and not from paired individual survey results.

## Discussion

Studies suggest that medical students and physicians are at a higher risk of depression as compared with the general population, and medical students experience a higher prevalence of anxiety than their non-medical peers in higher education [6-8,25-27,32]. This puts students at risk for other mental health difficulties and increases the risk of suicide. Medical schools across the United States are working to tackle this issue through prevention, intervention, and postvention efforts. Accreditation boards require medical schools to provide mental health support, such as counseling, access to 24/7 crisis intervention, and documented strategies to help medical students maintain and improve their overall health and wellness [33]. Even so, there continues to be high rates of burnout and diminished mental health among medical students over the course of their medical education. The aim of this study was to assess the impact of a mobile application, MindfulMEDS, designed specifically for the mental health and wellness needs of medical students. The purpose of this study was to assess the process of implementing the MindfulMEDS application with medical students. We were also interested in themes of student resiliency, mental health stigma, and connectedness to the school community of those students who engaged with the app. Through a mixed-methods approach, we were able to gather both quantitative and qualitative data that informed the usability of the mental health app, as well as ways to further improve and develop the tool for this population going forward.

While we did not find a significant change in resiliency levels based on results from the BRS, we saw a significant decrease in the perception of mental health stigma associated with utilizing mental health resources. It is notable that this decrease was observed based on one specific survey question between groups, not from paired individual surveys. Attrition rates and other personal and environmental factors that we did not control for in this study are important to consider when interpreting this data on stigma, but even with the limitations it is relevant as a point of discussion. A challenge educators face in this effort is a lack of awareness of the best approach to remove societal stigma towards seeking help [34]. As such, we hoped that rolling out a mental health and wellness tool for all students with the public support of faculty and administration would both normalize and promote mental wellness. Survey results showed that we met this goal and feedback from both focus groups also indicated that students are more likely to use a mental health resource if regular reminders and notifications are delivered. While we do not have specific information on whether students actually connected with the mental health resources provided, it does suggest that they were viewing the resource pages and presented with an opportunity for immediate support with the click of a button. An app that offers students a confidential and private means to access mental health care and information on an instantaneous basis would allow more students to seek treatment and services [35].

Also of note is the high percentage of “Get Help Now” clicks as compared with other parts of the app. We found this to be particularly informative in considering the future benefit of an app like MindfulMEDS for this population. This strongly suggests the need and interest in seeking support services through this platform. As we fine-tune and adjust the app going forward, it will be important to keep resources updated and expand as needed. There are times throughout the school year that we know tend to bring more stress and higher numbers of students accessing on-campus mental health counseling. For example, during spring and early summer (when the third surveys of this study were distributed), OMS II students are preparing for their board exams and tend to experience higher levels of stress and burnout. While we cannot infer causation, there was a relationship between this timing and a significant decrease in the percentage of OMS II students who reported that they disagree with the statement “I tend to take a long time to recover from setbacks in my life.” This suggests that students might benefit from more reminders of the resource during that time, and in general, that a more custom approach to the feed depending on both VCOM classification and context may be beneficial.

In considering a customized approach for medical students, engagement strategies will be important to increase interest and motivation to try a new resource. Focus group members discussed increasing notifications and regular communication to help draw students back into the app. Things like the technology issues and difficulty with navigation reported by focus groups may deter users from returning to the app. This feedback was useful in helping the team troubleshoot and make the app more user-friendly. For example, we changed some of the languages in the app to be more direct and easier to follow ("Mental Health Literacy" topics were previously labeled as "Heal".)

Further, we learned from the focus groups that some students used the app with their patients during clinical rotations. This “train-the-trainer” experience was an unexpected outcome and valuable in considering future implications for this app. This utilization suggests that there may be a further reach to spreading awareness about the importance of mental health and sharing evidence-based tools and information, and, importantly, it contributes to the overarching goals of reducing stigma and increasing access to mental health resources.

Limitations

There were limitations to the study that should be considered. First, there was attrition among initial, middle, and final survey distributions, which resulted in a small sample size. Information gathered in the surveys included measures of resiliency, connectedness to mental health resources, and stigma. Therefore, generalizability is limited in those areas. Usage data were gathered for all registered users, which provided a broader picture and helped to offset the limited survey participation. The second limitation was that we were not able to follow the course of use for individual participants as we did not track individual data. We did not have data on the frequency or duration of individual use, which is important to consider when looking at the usage data presented in this paper. More specific information about how students interact with the app over time would be helpful in determining the timing of notifications, adding certain types of information to the feed, and general patterns of use. It could also provide information about students discontinuing the use of the app.

Finally, the method by which an institution or organization promotes the utilization of an app is important. In this study, we did not take an aggressive approach to promoting the app. Research has shown that social influence and preconceptions towards the topic of mental health overall can overshadow the key components of any app [36]. It could be that some students see the app as beneficial, while others could express concern about the lack of quality content or how it will be perceived by their peers. In order to reduce barriers to adoption, we opted for self-discovery by those drawn to the subject matter. Although we believe it is best for this population to feel empowered to seek helpful mental health tools on their own, we learned that successful implementation requires a balanced approach between self-discovery and ongoing reminders of what tools are available. The app should be marketed carefully and allow the students to feel empowered about seeking help. This may be of particular importance for medical students given their high level of perfectionism and need for success, which has been shown to impact mental health and contribute towards the development of imposter syndrome [8]. Going forward we will continue to seek a balanced outreach approach to our medical student population to ensure we maximize utilization by those who may be seeking mental health support. School administration and faculty members have endorsed full support of this resource and have been informed on how to refer students to the app and other available mental health resources as appropriate.

Future research

The user activity data reflect MindfulMEDS improved engagement with mental health protective resources with a noted increase in shared protective factors: suicide prevention (SP), mindfulness-based stress reduction (MBSR), mental health literacy (MHL), and disordered eating prevention (DEP) content. Future research linking the impact of this content to the mental health outcomes of students through pre- and post-test mental health screening would be a logical next step to determine the impact. In addition, since our focus group interactions helped us understand the usefulness of the app in clinical sessions with patients, measuring how the app increases compliance and mental health literacy among physicians and patients would be another compelling area of research.

Further, the data shows that users consumed a noteworthy amount of content in the areas of mental health literacy, social-emotional learning, mindfulness-based stress reduction, and suicide prevention. It was of note that there was a greater use of shorter modules in the feed versus the longer modules in the library. This could have been a matter of convenience or could highlight how medical students prefer to consume mental health content. In future research, we would like to explore these findings more deeply and integrate other tools and/or metrics that would allow for a more complex investigation of a user’s level of resiliency, based on the available evidence-based protective factors.

## Conclusions

Efforts to tackle the elevated levels of burnout, depression, and suicidality in medical students and physicians are ongoing. Increasing awareness and prevention early in medical training should be integral in both curricular activities and embedded in the culture of each medical school. We aimed to show that the use of mobile-based technology designed specifically for the mental health and wellness needs of medical students, MindfulMEDS, would be one way to bridge the gap between existing mental health resources. We learned that students found value in this app, utilized mental health screeners embedded within the app, and, perhaps most importantly, used the app content to increase their own mental health literacy and unexpectedly the mental health literacy of patients. To this end, results show the feasibility of the app and also provide valuable information about what medical students find useful in this type of app. As we continue to look for ways of decreasing the stigma around seeking mental health support, we believe that incorporating an app like MindfulMEDS into existing student resources in medical schools may be an important step. Data from this study shows that the medical students clicked on buttons related to crisis support and local mental health resources. That data alone suggests that we are tapping into a need for more support and resources in this area, and students used the app as a means of accessing that support. Furthermore, students valued peer-to-peer content such as video interviews with other medical students and faculty, which helped normalize the struggles of being a medical student and promoted connectedness to their academic community. Prevention, skill-building, and access to resources are all well-known to contribute to resiliency and also the building blocks for MindulMEDS, so we will continue to assess how the app can be integrated into existing programming for student health and wellness. Ongoing expansion and modification of best practices to promote the mental health of physicians-in-training are important on an individual level for students, and on a macro level, it will impact future patients and hopefully contribute to a more humanistic model of medicine overall.
